# Peroxides with Anthelmintic, Antiprotozoal, Fungicidal and Antiviral Bioactivity: Properties, Synthesis and Reactions

**DOI:** 10.3390/molecules22111881

**Published:** 2017-11-02

**Authors:** Vera A. Vil’, Ivan A. Yaremenko, Alexey I. Ilovaisky, Alexander O. Terent’ev

**Affiliations:** 1 N. D. Zelinsky Institute of Organic Chemistry, Russian Academy of Sciences, 47 Leninsky Prospekt, 119991 Moscow, Russia; vil@ioc.ac.ru (V.A.V.); yaremenko@ioc.ac.ru (I.A.Y.); ilov@ioc.ac.ru (A.I.I.); 2 Faculty of Chemical and Pharmaceutical Technology and Biomedical Products, D. I. Mendeleev University of Chemical Technology of Russia, 9 Miusskaya Square, 125047 Moscow, Russia; 3 All-Russian Research Institute for Phytopathology, B. Vyazyomy, 143050 Moscow, Russia

**Keywords:** peroxides, anthelmintic, antiprotozoal, fungicidal, antiviral

## Abstract

The biological activity of organic peroxides is usually associated with the antimalarial properties of artemisinin and its derivatives. However, the analysis of published data indicates that organic peroxides exhibit a variety of biological activity, which is still being given insufficient attention. In the present review, we deal with natural, semi-synthetic and synthetic peroxides exhibiting anthelmintic, antiprotozoal, fungicidal, antiviral and other activities that have not been described in detail earlier. The review is mainly concerned with the development of methods for the synthesis of biologically active natural peroxides, as well as its isolation from natural sources and the modification of natural peroxides. In addition, much attention is paid to the substantially cheaper biologically active synthetic peroxides. The present review summarizes 217 publications mainly from 2000 onwards.

## 1. Introduction

Peroxides are widely used in various areas of life [1,2,3]. Traditional and the most developed field is the application of peroxides as radical initiators in industrial processes in the manufacture of polymers from unsaturated monomers: styrenes, butadienes, chlorovinyls, ethylenes, acrylates, as well as in crosslinking of silicone rubbers, acrylonitrile-butadiene rubbers, fluororubbers, polyethylene, ethylene-propylene copolymer, etc. [4,5,6,7,8,9].

Hydrogen peroxide and peracids are active components of antiseptics and disinfectants [10,11,12,13,14]. Synthesis and mechanism of antiseptic action of hydrogen peroxide and the most common peracids (performic, peracetic, etc.) are elucidated in a few studies [15,16,17] and are not considered in this review.

**Figure 1:**
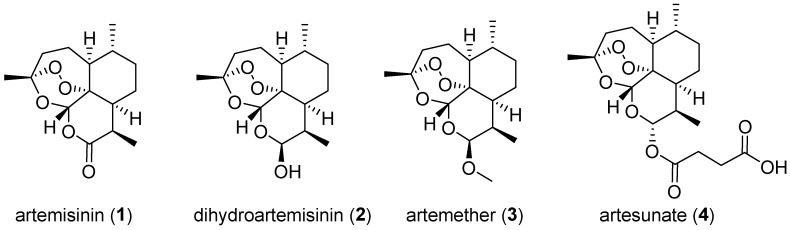
Artemisinin and its semi-synthetic derivatives.

Antimalarial properties of peroxides are currently intensively studied. Artemisinin (Qinghaosu) (**1**), a natural peroxide possessing high antimalarial activity, was isolated in 1971 from leaves of annual wormwood (*Artemisia annua*) in the context of the scientific program “Project 523”, initiated by Chinese government in 1967 [18,19,20]. The Nobel Prize in Physiology or Medicine 2015 was awarded to Chinese pharmaceutical chemist Tu Youyou “for her discoveries concerning a novel therapy against Malaria” [21,22,23]. Considering the development of antibiotic resistance of Plasmodium to some traditional drugs, such as quinine, chloroquine, and mefloquine, and other anti-parasitic ones, pharmaceuticals based on artemisinin and its semi-synthetic derivatives—dihydroartemisinin (**2**), artemether (**3**) and artesunate (**4**) (Figure 1)—are currently the most effective drugs against malaria [24,25,26,27,28,29,30].

The modern trend in medicinal chemistry of peroxides is the search of effective anticancer drugs. The natural and synthetic peroxides exhibiting a cytotoxic effect on cancer cells already include hundreds of compounds [31,32,33,34]. Peroxides possessing antimalarial and cytotoxic activity are the subject of numerous studies [35,36,37,38,39,40,41,42], and are not considered in this review.

In the present review, we deal with natural, semi-synthetic and synthetic peroxides exhibiting anthelmintic, antiprotozoal, fungicidal, antiviral and other activities that have not been described in detail earlier. The review is mainly concerned with the synthesis of such peroxides, as well as its isolation from natural sources and covers literature published between 1912 and 2017.

There are several review articles, where the various kinds of the biological activity of artemisinin [43,44,45] and artemether [46,47]; the problems of trematode infection therapy with artemisinin, its derivatives and several synthetic ozonides [48]; and antiviral activity of artemisinin and artesunate [49] are discussed. Advances in the development of anti-parasitic peroxides are described in the review of Muraleedharan [50]. A number of reviews are devoted to promising anthelmintic peroxides [51]. Some natural antiviral peroxides are mentioned in the review [52]. However, none of these articles pay sufficient attention to the methods of peroxide synthesis.

**Figure 2:**
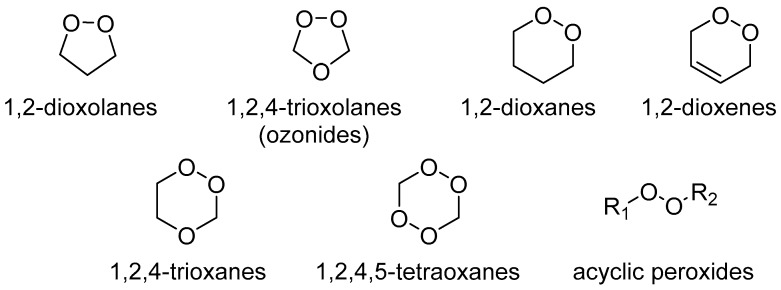
Reviewed cyclic and acyclic peroxides.

Since peroxides with a related structure have different types of activity, the systematization of this review is based on the structure of the peroxide fragment (Figure 2). The first sections consider the preparation of cyclic peroxides in order of increasing cycle and the number of oxygen atoms in it, while the last section deals with peroxides of acyclic structure. The following abbreviations are used in describing the biological activity of peroxides: minimum inhibitory concentration (MIC), minimum lethal concentration (MLC), half maximal inhibitory concentration (IC_50_), concentration of inhibiting 90% of activity (IC_90_), half maximal effective concentration (ЕС_50_), and median effective dose (ED_50_) [53,54].

## 2. 1,2-Dioxolanes

**Scheme 1:**
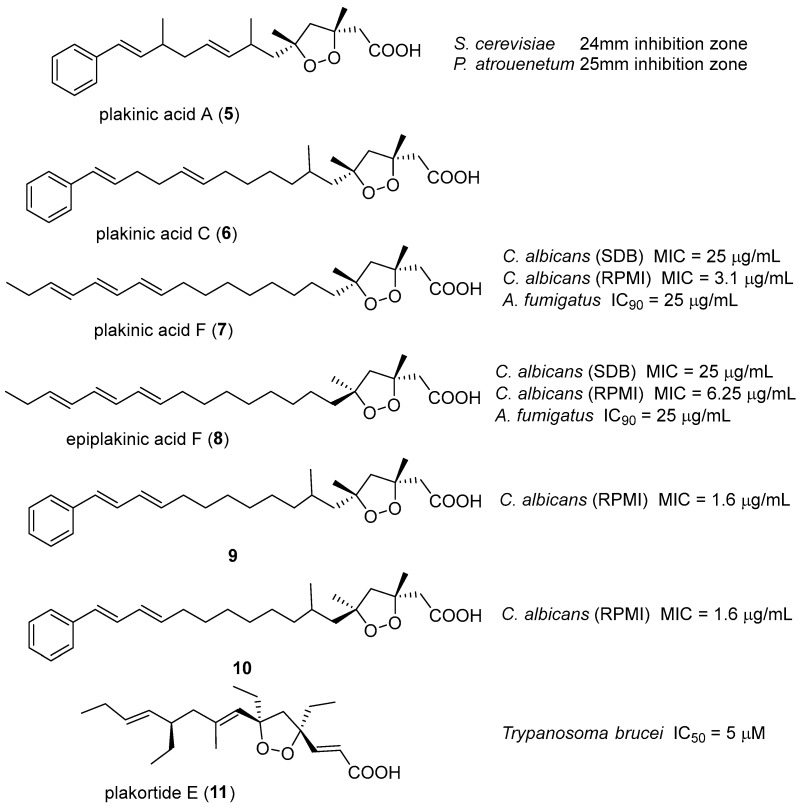
Antifungal and anti-parasitic activity of 1,2-dioxolanes, isolated from the sponges *Plakinidae*.

A number of cyclic peroxides, many of which exhibit antibacterial, antifungal and anti-cancer activity, were isolated from the marine organisms, in particular from the sponges *Plakinidae* [55]. Plakinic acid А (**5**) effectively inhibits the growth of fungi *Saccharomyces cerevisiae* and *Penicillium atrounenetum* [56]; plakinic acid F (**7**) and *epi*-plakinic acid F (**8**) exhibit moderate antifungal activity against *Candida albicans* and *Aspergillus fumigatus* [57]; 1,2-dioxolane acids **9** and **10** inhibit the growth of *Candida albicans* [58]; and plakortide Е (**11**) show good activity against *Trypanosoma brucei* (Scheme 1) [59].

**Scheme 2:**
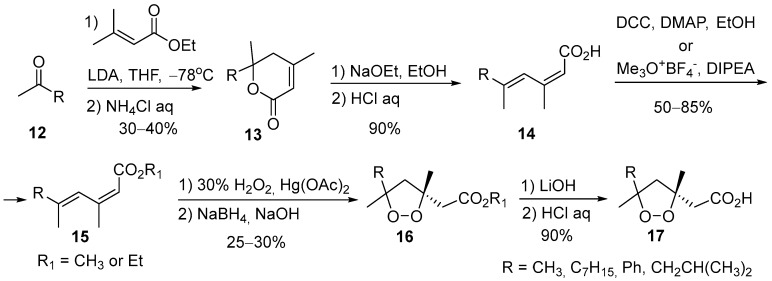
The first synthesis of saturated analogs of plakinic acids **17**.

The first synthesis of diastereomeric saturated analogs of plakinic acids A, C and D **17** was reported in 1996 by Bloodworth and colleagues [60]. Peroxides **17** were prepared in four steps from ketones **12**. In the first step, ketone **12** was condensed with ethyl 3-methylbut-2-enoate with formation cyclic lactones **13** hydrolysis, which led to acids **14**. Peroxymercuration of esters **15** followed by reduction with sodium borohydride afforded 1,2-dioxolanes **16** saponification, which resulted in 1,2-dioxolanes **17** with carboxylic group (Scheme 2).

**Scheme 3:**
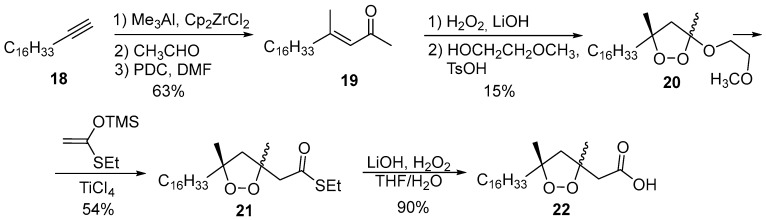
The synthesis of diastereomeric analogs of plakinic acids **22**.

The synthesis of diastereomeric 1,2-dioxolanes **22** from alkynes was described [61]. Carboalumination of alkyne **18**, followed by treatment of the intermediate alkenylaluminum with acetaldehyde, resulted in an allylic alcohol that was oxidized to enone **19**. Conjugate addition of H_2_O_2_ to **19** in the presence of LiOH followed by acid-catalyzed esterification of diastereomeric dioxinole by 2-methoxyethanol provided alkoxydioxolane **20**. Substitution of the methoxyethoxy group in **20** by the action of silyl keteneacetal ethyl thioacetal in the presence of TiCl_4_ led to thioether **21** as a 1:1 mixture of two diastereomers. The hardly-separable *cis*- and *trans*-diastereomeric peroxides **22** were obtained as a result of hydrolysis with high yield (Scheme 3).

**Scheme 4:**
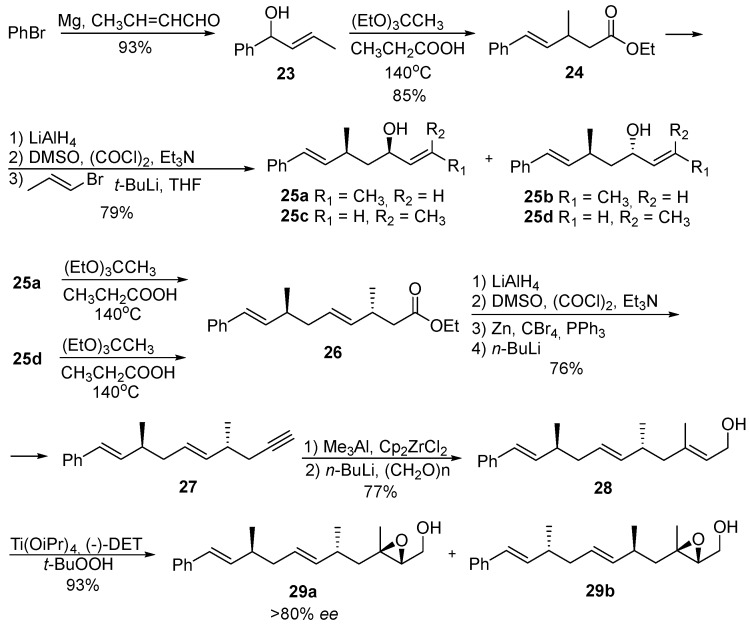
Construction of asymmetric plakinic acids side chain.

In 2006, the first asymmetric synthesis of plakinic acids was reported [62]. The key step of side chain construction was synthesis of diastereomeric allylic alcohols **25** from bromobenzene. This transformation included subsequent addition Mg-organic compound to crotonaldehyde with formation of alcohol **23**, Claisen homologation resulted in ester **24**, and reaction of an intermediate aldehyde with propenyl lithium (Scheme 4).

**Scheme 5:**
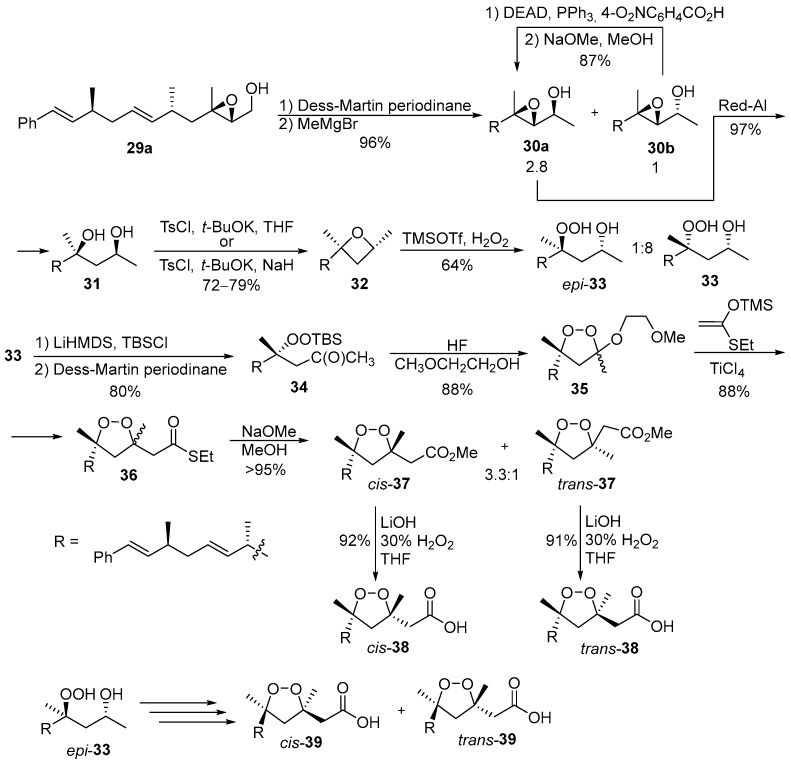
Asymmetric synthesis of plakinic acids **38** and **39**.

As a result of following transformation epoxy alcohol **29a** was prepared, which was oxidized into aldehyde; subsequent addition of methylmagnesium bromide resulted in isomeric secondary epoxy alcohols **30а** and **30b** (Scheme 5). After transformation of minor **30b** into **30a**, the latter was reduced to diol **31**. The treatment of diol **31** with stoichiometric quantity of TsCl and excess of *t*-BuOK led to oxetane **32**. The ring opening with TMSOTf provided easily separable 3-hydroxy hydroperoxides **33** and *epi*-**33**; **33** was converted into peroxy ketone **34** by subsequent silylation and oxidation. The ketone **34** was transformed into alkoxydioxolane **35**, and after that into thioester **36**. The desirable plakinic acids **38** were prepared by hydrolysis of methyl esters **37**. A similar strategy was used for the synthesis of stereomeric acids **39** from 3-hydroxy hydroperoxide *epi*-**33**.

**Scheme 6:**
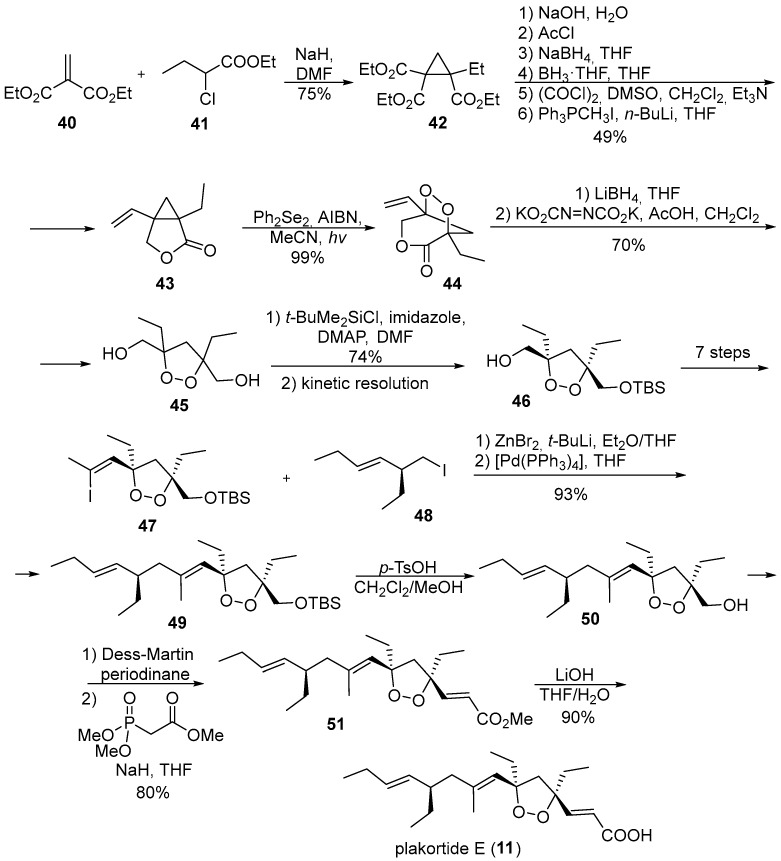
Synthesis of plakortide E (**11**) by radical oxygenation of cyclopropanes.

A total synthesis of plakortide E (**11**) based on radical oxygenation of vinylcyclopropanes was reported [63,64]. The key intermediate 2-ethyl-1,1,2-cyclopropanetricarboxylate (**42**) prepared from methylene malonate (**40**) and α-chloro ester **41** was transformed into lactone **43**. The treatment of the latter with oxygen, Ph_2_Se_2_ and AIBN furnished spiro 1,2-dioxolane **44**, followed by lactone ring opening of **44** to form diol **45**. The precursor of the plakortide E **49** was synthesized from the iodo-derivative of 1,2-dioxolane **47** and the halogen derivative **48** by Negishi reaction. Desilylation of alcohol **49**, subsequent oxidation and Horner–Wadsworth–Emmons olefination provided ester **51** which was hydrolyzed with formation of plakortide E (**11**) (Scheme 6) [63].

**Scheme 7:**
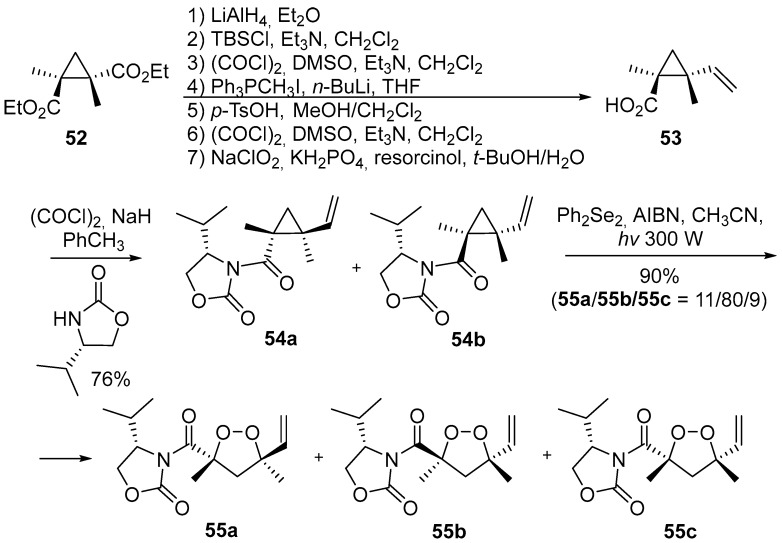
Construction of 1,2-dioxolane fragment for epiplakinic acid F (**8**).

A similar strategy based on the radical oxygenation of vinyl cyclopropanes was used for the synthesis of epiplakinic acid F (**8**) [65]. The vinyl cyclopropane **53** obtained from *trans*-1,2-cyclopropanedicarboxylate **52**, was then converted to 1,2-dioxolane **55** (Scheme 7). After separation of the diastereomeric mixture isomer **55b** was used for further transformations.

**Scheme 8:**
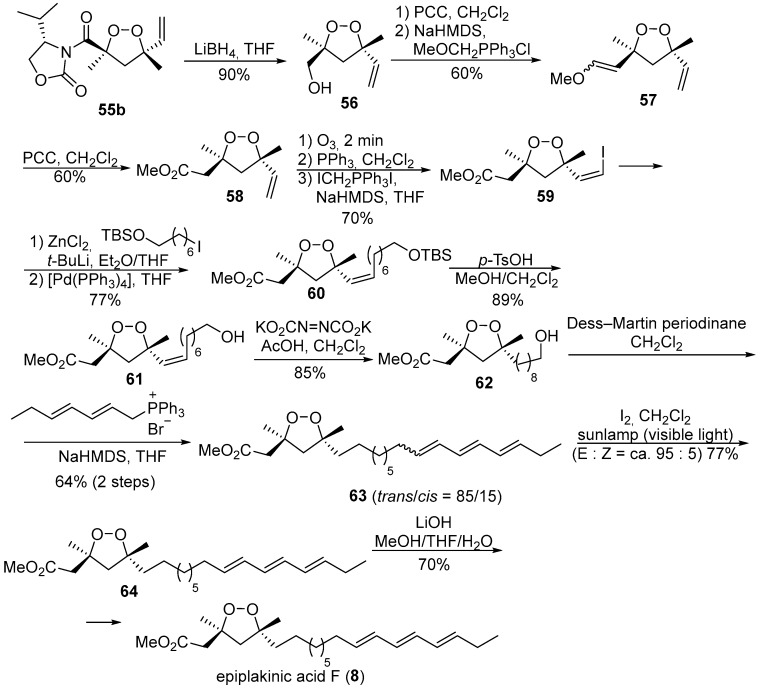
Asymmetric synthesis of epiplakinic acid F (**8**).

Enantiomerically pure **55b** was reduced by LiBH_4_ to alcohol **56**, which was transformed to vinylether **57** by subsequent PCC oxidation and Wittig olefination. Oxidation of **57** to methyl ester **58**, reductive ozonolysis of **58**, and Wittig olefination gave predominantly the Z-isomer of vinyl iodide **59**. The Negishi coupling of **59** with the halogen derivative led to the desired product **60**. Desilylation of **60** followed by reduction of **61** resulted in saturated alcohol **62**. The oxidation of **62** led to an aldehyde, the Wittig olefination of which provided the precursor **63** as a mixture of isomers. The product of photoinduced isomerization of this mixture was the *trans*-isomer **64**. The target epiplakinic acid F (**8**) was obtained by alkaline hydrolysis of ester **64** (Scheme 8).

**Scheme 9:**
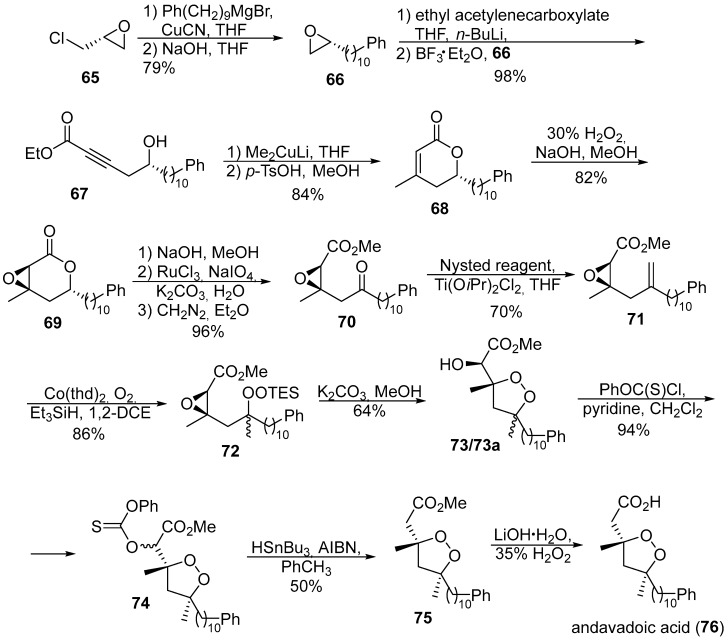
Synthesis of andavadoic acid (**76**).

The method of the preparation of andavadoic acid (**76**), a natural compound isolated from the sponges of *Plaxortis aff simplex*, based on the Isayama−Mukaiyama reaction and subsequent cyclization was known [66]. The starting substrate was epichlorohydrin (**65**), which was converted to epoxide **66** by a subsequent the organomagnesium compound addition and cyclization with the help of alkaline. The regioselective opening of the epoxide cycle of **66** by the lithium salt of ethyl propiolate in the presence of BF_3_ resulted in the secondary alcohol **67** in almost quantitative yield. Alcohol **67** was converted into lactone **68** by reaction with Me_2_CuLi, followed by acidification. Oxidation of lactone **68** to **69**, subsequent ring opening, oxidation of hydroxyl to the carbonyl group, and methylation resulted in epoxy ketone **70**, which was then converted to epoxy alkene **71**. Isayama–Mukaiyama peroxidation, the base-catalyzed cyclization of peroxide **72**, leading to a mixture of diastereomeric 1,2-dioxolanes **73**/**73a** followed by separation and thioacylation resulted in a peroxy thioester **74** which was converted into andavadoic acid (**76**) by subsequent reduction and hydrolysis of ester **75** (Scheme 9).

**Scheme 10:**
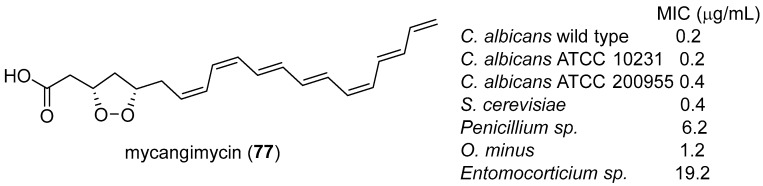
Natural peroxide mycangimycin and its fungicidal activity.

The symbiont of beetle of the southern pine (*Dendroctonus frontalis*), actinomycetous bacterium produces mycangimycin peroxide (**77**) with pronounced fungicidal activity (Scheme 10) [67]. Mycangimycin effectively inhibits growth of *Candida albicans* wild type, *C. albicans* ATCC10231, amphotericin-resistant strain *C. albicans* ATCC 200955, *Saccharomyces cerevisiae* and *Ophiostoma minus* [68].

**Scheme 11:**
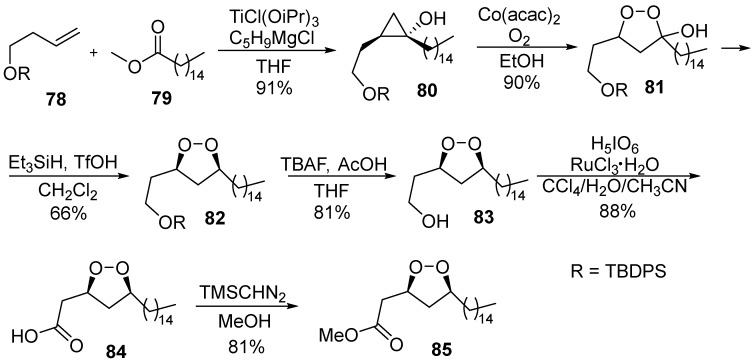
The synthesis of saturated analogs of mycangimycin **84** and **85**.

Two saturated analogs of mycangimycin were synthesized from alkene **78** and ester **79** (Scheme 11) [69]. The Kulinkovich reaction of **78** with **79** resulted in cyclopropane **80**, which formed 1,2-dioxolane **81** by a cobalt-catalyzed cleavage in the presence of oxygen. TfOH-catalyzed reduction of the alcohol **81** by silane led to 3,5-disubstituted 1,2-dioxolane **82**. The result of desilylation and oxidation of the obtained alcohol **83** is the first saturated analog of mycangimycin, acid **84**, which can be converted into ester **85**.

**Scheme 12:**
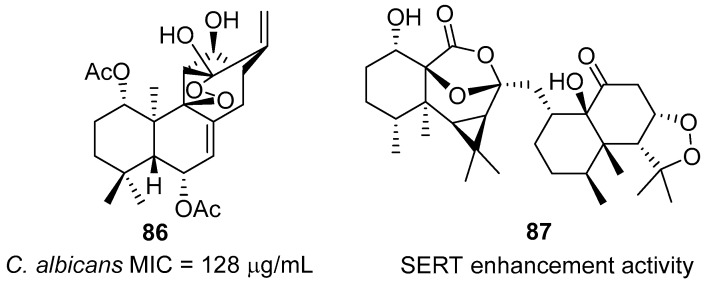
Terpene peroxides **86** and **87**.

The diterpenoid peroxide **86**, which has a weak fungicidal activity against *C. albicans*, was isolated from the liverwort *Jungermannia atrobrunnea* (Scheme 12) [70]. Dinardokanshone B (**87**), sesquiterpene peroxide, isolated from the roots and rhizomes of *Nardostachys chinensis Batal.* (Valerianaceae) showed significant enhancement effects on SERT activity (Scheme 12) [71].

**Scheme 13:**
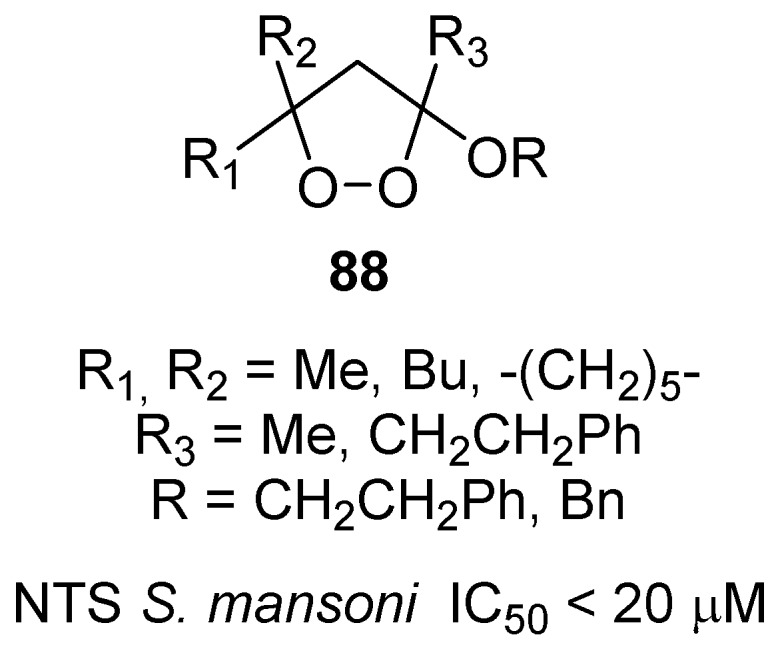
1,2-Dioxolanes **88** exhibiting high activity against schistosomiasis.

Synthetic 1,2-dioxolanes **88** showed high in vitro activity against helminths *Schistosoma mansoni* (Scheme 13) [72].

**Scheme 14:**
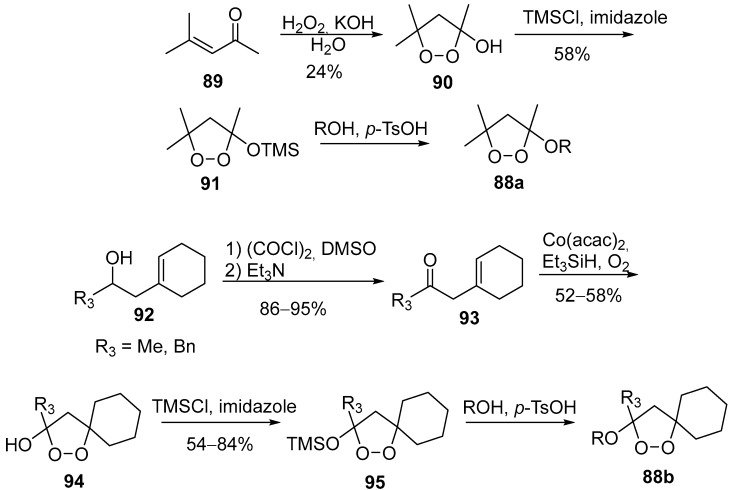
Synthesis of anthelmintic 1,2-dioxolanes **88**.

5,5-Dimethyl 1,2-dioxolanes **88a** were prepared by peroxidation of mesityl oxide (**89**) in the basic medium, followed by alkylation of the free hydroxyl group of 3-hydroxy-1,2-dioxolanes **90** (Scheme 14) [73]. Synthesis of cyclohexyl derivatives **88b** started from oxidation of alcohol **92** to an unsaturated ketone **93**, followed by Isayama–Mukaiyama peroxidation with formation of 1,2-dioxolane **94**. Further protection of hydroxyl group resulted in derivatives **88b** (Scheme 14) [73].

**Scheme 15:**
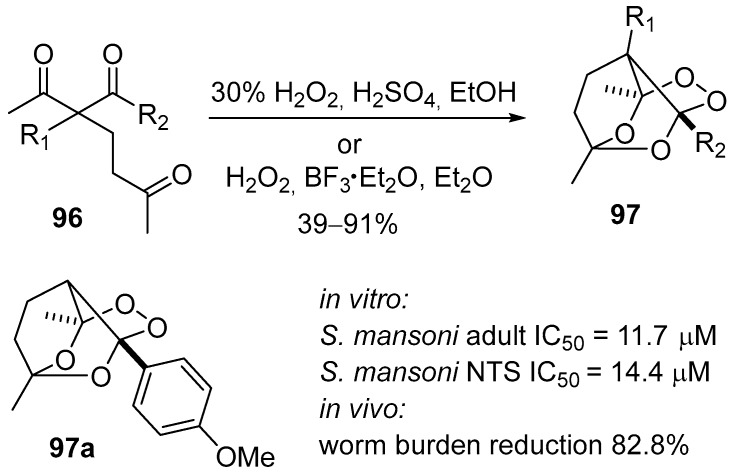
Synthesis of tricyclic monoperoxides **97** with high anti-schistosomal activity.

The series of tricyclic monoperoxides **97** with 1,2-dioxolane moiety showed a high anti-schistosomal activity. The maximum activity was observed for compound **97a**, which revealed high worm burden reductions by 82.8% in *S. mansoni* mouse model (Scheme 15) [74]. Peroxides **97** can be obtained from β, *δ*-triketones **96** and hydrogen peroxide with use of either sulfuric acid [75], or boron trifluoride [76] as catalyst and co-solvent (Scheme 15).

## 3. 1,2,4-Trioxolanes (Ozonides)

**Scheme 16:**
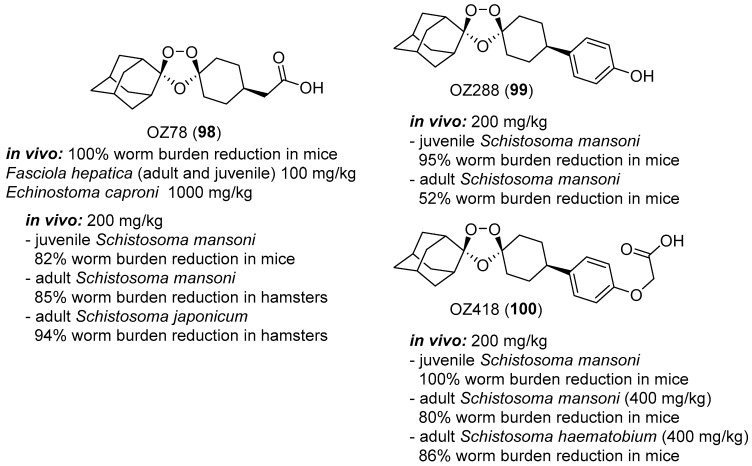
Tetrasubstituted synthetic ozonides with the highest anthelmintic activity.

Various tetrasubstituted 1,2,4-trioxolanes (ozonides) have become the breakthrough in the field of biologically active synthetic peroxides. Nowadays ozonides are considered as the most promising candidates in the treatment of helminth diseases (Scheme 16). The 100% worm burden reductions were observed with a single oral dose of 1000 mg/kg OZ78 (**98**) in *Echinostoma caproni*-infected mice. A single dose of 100 mg/kg OZ78 (**98**) resulted in 100% worm burden reductions against juvenile and adult *Fasciola hepatica*-mouse model [77]. Single oral doses of 100 mg/kg OZ78 (**98**) is efficient against adult triclabendazole-resistant *F. hepatica*-infected rats [78]. Later, the efficacy of 1,2,4-trioxolane OZ78 (**98**) against an experimental infection with *Fasciola hepatica* in sheep was confirmed [79,80]. It was found that the spiroadamantane fragment and the carboxyl group in the OZ78 (**98**) are necessary for activity against *F. hepatica* [81].

The ozonides OZ78 (**98**) and OZ288 (**99**) showed low toxicity and high efficiency against helminth cultures *Schistosoma mansoni* and *S. japonicum* harboured in mice and hamsters with a single oral dose of 200 mg/kg [82,83]. Antichistosomal activity of OZ78 (**98**) against *S. japonicum* was confirmed in experiments in mice and rabbits [84]. Later, the promising ozonide OZ418 (**100**) with high activity against both helminths of *S. mansoni* and *S. haematobium* was discovered [85].

**Scheme 17:**
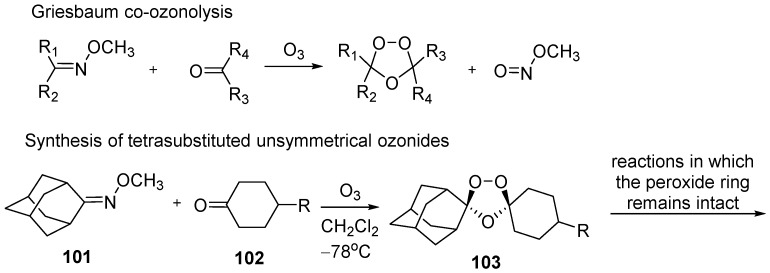
Synthesis of tetrasubstituted unsymmetrical ozonides.

Synthesis of tetrasubstituted unsymmetrical ozonides was discovered by Griesbaum and colleagues in 1995 (Scheme 17, top) [86,87]. Later this method was used for the diastereoselective synthesis of tetrasubstituted ozonides **103**. Peroxides **103** were prepared by ozonolysis of 2-adamantanone O-methyl oxime (**101**) in the presence of substituted cyclohexanones **102** (Scheme 17, bottom) [88,89]. 1,2,4-Trioxolane ring in compounds **103** is resistant to the action of a wide range of reagents, which allows to proceed a variety of modifications of the cyclohexane substituent [88].

## 4. 1,2-Dioxanes

**Scheme 18:**
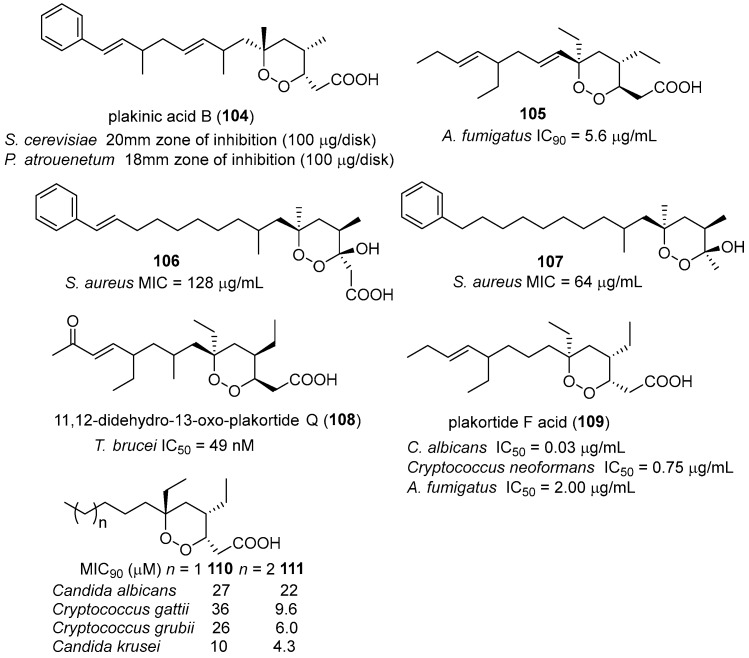
1,2-Dioxanes isolated from the sponges *Plakinidae*; their antifungal, antibacterial and anti-trypanosomal activity is also shown.

A number of compounds containing 1,2-dioxane fragment isolated from the *Plakinidae* sponges showed high fungicidal and anti-trypanosomal activity. Plakinic acid B (**104**) exhibited good fungicidal activity against *Saccharomyces cerevisiae* and *Penicillium atrovenetum* (Scheme 18) [56]. The acid **105** in vitro inhibits the growth of fungi *Aspergillus fumigatus* with IC_90_ = 5.6 µg/mL [58]. Compounds **106** and **107** were weakly active against *Staphylococcus aureus* [90]. 11,12-Didehydro-13-oxo-plakortide Q (**108**) was more active against *Trypanosoma brucei* [91]. Plakortide F acid (PFA) (**109**) showed high activity against *Candida albicans*, *Cryptococcus neoformans* and *A. fumigatus* [21]. Plakortides **110** and **111** also demonstrated high activity against a number of fungi [92]. Significant fungicidal activity of mixture of peroxyketal acids isolated from sea sponges was also reported [93].

**Scheme 19:**
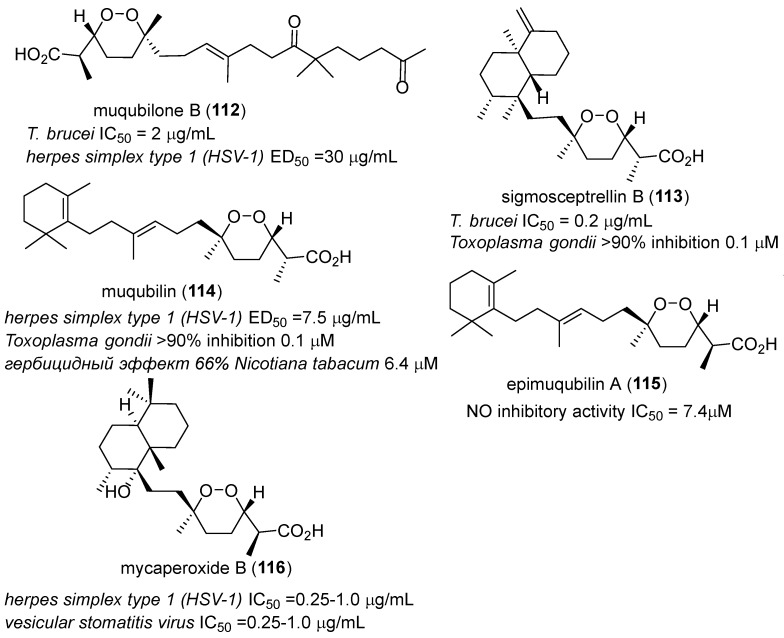
Muqubilin (**114**) and related natural peroxides.

A series of peroxyterpenes was isolated from the sponges *Diacarnus bismarckensis*, the most active of which, (+)-muqubilone B (**112**) and sigmosceptrellin B (**113**) showed high activity against *Trypanosoma brucei* [94]. (+)-Muqubilone B (**112**) and muqubilin (**114**) (isolated from the sponges *Prianos* [95]) were in vitro effective against herpes virus (HSV-1), muqubilin (**114**) and (−)-sigmosceptrellin B (**113**) exhibited in vitro activity against *Toxoplasma gondii* (Scheme 19) [96]. Muqubilin (**114**) possesses herbicidal activity against tobacco *Nicotiana tabacum* [97], as well as epimuqubilin (**115**) showed NO inhibitory activity [98]. Mycaperoxide В (**116**) displays high antiviral activity against vesicular stomatitis virus and herpes simplex virus type-1 (HCV-1) [99].

**Scheme 20:**
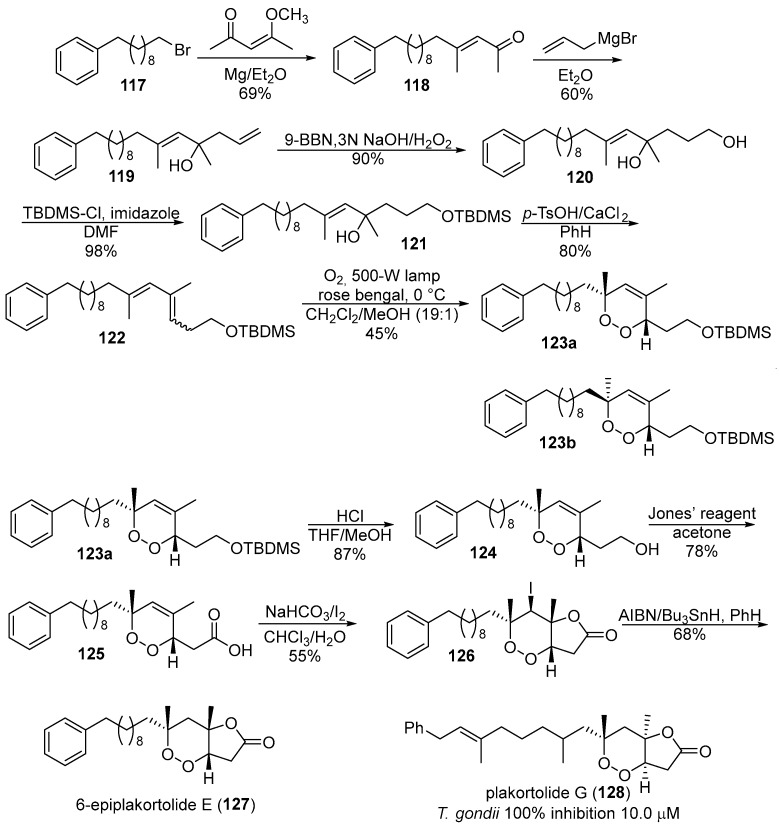
Synthesis of 6-epiplakortolide Е (**127**).

Several synthetic approaches to plakinic acids and their derivatives containing 1,2-dioxane ring were described. Natural 6-epiplakortolide E (**127**) was firstly synthesized from available 1-bromo-10-phenyldecane (**117**) in 10 steps by Diels–Alder reaction with singlet oxygen followed by iodolactonization (Scheme 20) [100]. In the first stage, the addition of the organomagnesium reagent to the unsaturated ketone resulted in enone **118**, which was converted to a tertiary alcohol **119**. Hydroboration of **119** led to diol **120**, protection of hydroxyl group of which provide silyl ether **121**. Diels–Alder reaction of diene **122** prepared by dehydration of **121** with singlet oxygen resulted in diastereomeric 1,2-dioxanes **123а** and **123b**. Deprotected diastereomer **124** was oxidized to acid **125**, subsequent iodolactonization of this acid gave bicycle **126**. Desirable 6-epiplakortolide E (**127**) was formed as result of radical reduction of iodine-containing bicycle **126**. It was noted that related plakortolide G (**128**) is active against the protozoa *Toxoplasma gondii* [101].

**Scheme 21:**
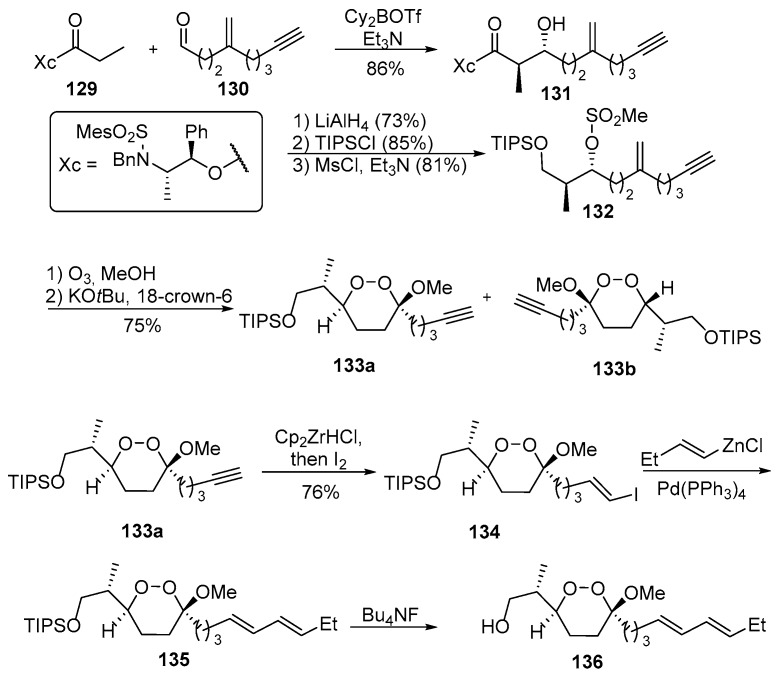
Asymmetric synthesis of 1,2-dioxane **136**.

Asymmetric synthesis of alkoxy-1,2-dioxane **136** related to plakortolides was realized in 8 steps from carbonyl compounds **129** and **130** (Scheme 21) [102]. Enantioselective aldol reaction in first step resulted in aldol **131** which was converted into protected diol **132** in three steps. Diastereomeric 1,2-dioxanes **133a** and **133b** were formed by selective ozonolysis of double bond of **132**. Hydrozirconation of **133а** with following treatment by iodine led to the formation of vinyl iodide **134**, followed cross-coupling of one resulted in 1,2-dioxane **135**. Deprotection of **135** provided desirable peroxide **136**.

**Scheme 22:**
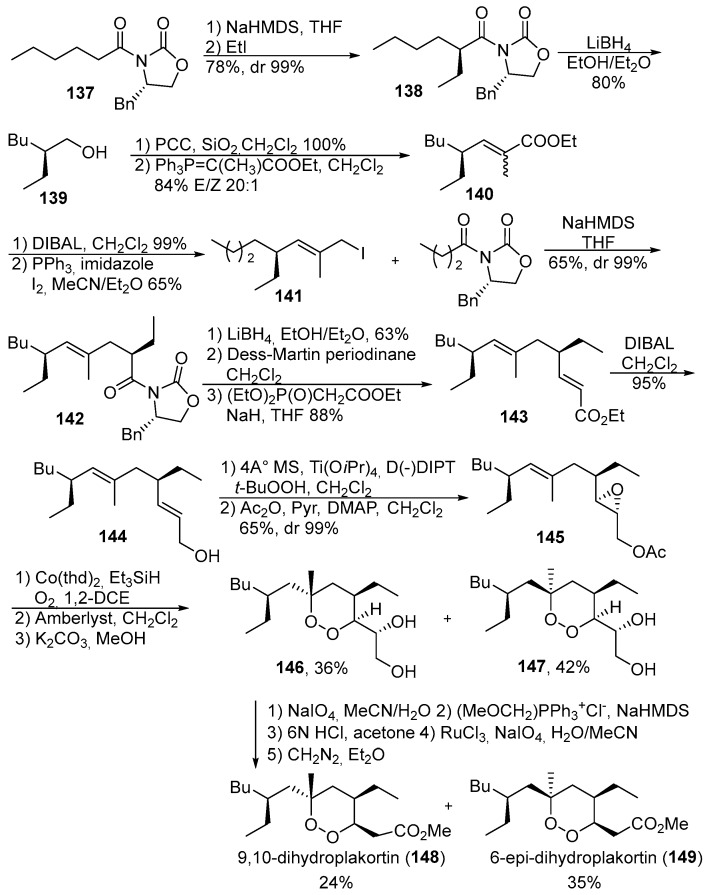
Synthesis of natural endoperoxide 9,10-dihydroplakortin (**148**) and its diastereomer **149**.

Natural endoperoxide 9,10-dihydroplakortin (**148**) and diastereomer **149** were synthesized through Evans’ chiral auxiliary chemistry (Scheme 22) [103]. Alkylation of oxazolidinone **137** and subsequent reduction of **138** led to alcohol **139**, which was transformed into acrylic ester **140** (predominantly in *E*-configuration) by oxidation and Horner–Wadsworth–Emmons olefination. The ester **140** was reduced into corresponding alcohol, which was converted into iodide **141**, alkylation of oxazolidinone by which resulted in derivative **142**. The cleavage and olefination of **142** provided the ester **143**. The reduction of **143** furnished alcohol **144** followed by Sharpless epoxidation and protection of primary hydroxy-group with formation of epoxide **145**. Isayama–Mukaiyama peroxidation of epoxide **145** afforded mixture of diastereomeric 1,2-dioxanes **146** and **147** which were independently transformed into 9,10-dihydroplakortin (**148**) and 6-*epi*-dihydroplakortin (**149**).

**Scheme 23:**
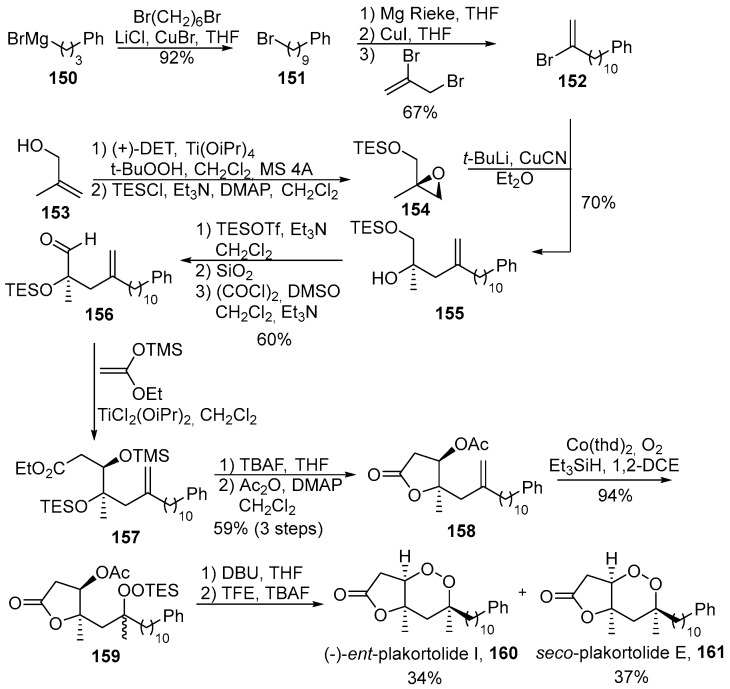
Synthesis of diastereomeric plakortolides **160** and **161**.

In the synthesis of diastereomeric plakortolides **160** and **161**, the key steps are construction of protected diol **155** from protected 2-methyl glycidol **154** and vinyl bromide **152**, diastereoselective Mukaiyama addition of aldehyde **156** with formation of ester **157** and hydroperoxidation of alkene **158** with subsequent cyclization of **159** (Scheme 23) [104].

**Scheme 24:**
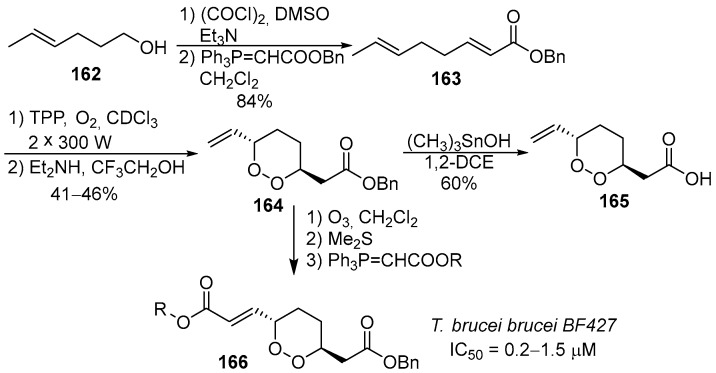
Synthesis of 1,2-dioxanes **166** with high anti-trypanosomal activity.

Synthesis of analogs of plakinic acids, 1,2-dioxanes **166** with a pronounced anti-trypanosomal activity was proposed from *E*-hexen-4-ol (**162**) (Scheme 24) [105]. Swern oxidation of alcohol **162** and subsequent Wittig-olefination led to ester **163**. The reaction of unsaturated scaffold **163** with singlet oxygen and following cyclization provided 1,2-dioxane **164**, which was hydrolyzed to acid **165**. Derivatives **166** were prepared from ester **164** by consistent ozonolysis, reductive cleavage and Wittig reaction. Compounds **166** exhibited high activity against *T. brucei brucei* BF427.

**Scheme 25:**
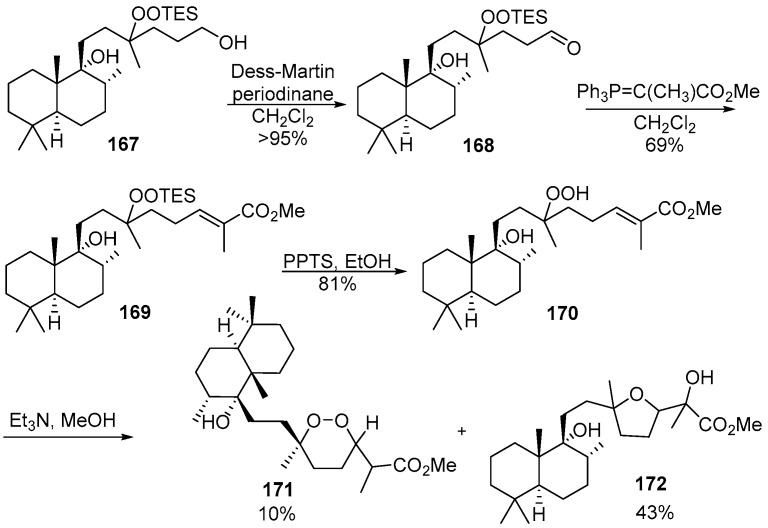
Synthesis of diastereomeric peroxides **171**.

Diastereomeric cyclic peroxides **171**, one of which is the methyl ester of the mycaperoxide B (**116**), were prepared from protected hydroxyperoxide **167** (Scheme 25) [106]. In the first step, peroxide **167** was oxidized to aldehyde **168**, which was converted into unsaturated ester **169** by Wittig reaction. Hydroperoxide **170** synthesized by deprotection of **169** was cyclized into 1,2-dioxane **171** and oxolane **172** by the action of triethylamine.

## 5. 1,2-Dioxenes

**Scheme 26:**
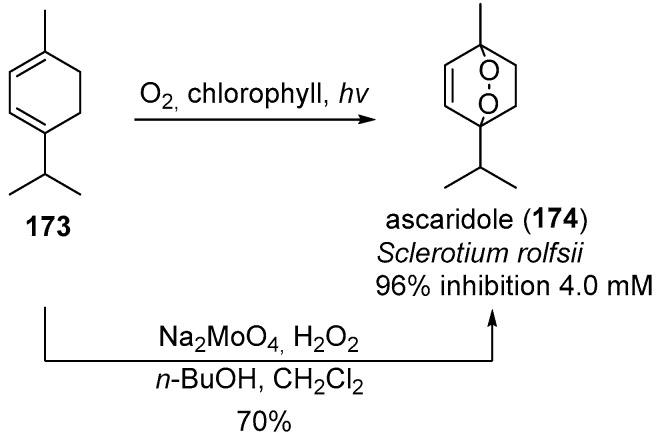
Ascaridole synthesis (**174**).

The plant *Chenopodium ambrosioides* is used for the production of essential chenopodium oil, which has been used as an anthelmintic agent for a long time [107,108]. The first isolation of the most active component-ascaridole (**174**), from the *Chenopodium ambrosioides* and the determination of its structure was dated to the beginning of the last century [109,110,111,112]. In the 1950s, the ascaridole (**174**) was completely characterized (Scheme 26) [113,114].

The first laboratory synthesis of ascaridole (**174**) was performed via photo-induced addition of singlet oxygen to terpene **173** by Schenck in 1944 (Scheme 26) [115]. Later, this reaction was realized in an industrial scale, because ascaridole was of great importance as an anthelmintic agent [116]. The method for the preparation of ascaridole using singlet oxygen generated in situ from sodium molybdate and hydrogen peroxide is known [117].

It was shown that ascaridole (**174**) at concentration 4 mM almost completely inhibits the growth of fungi *Sclerotium rolfsii* [118]. Presently, the side effects of ascaridole on the gastrointestinal tract have been described, and ascaridole is currently not used [119].

**Scheme 27:**
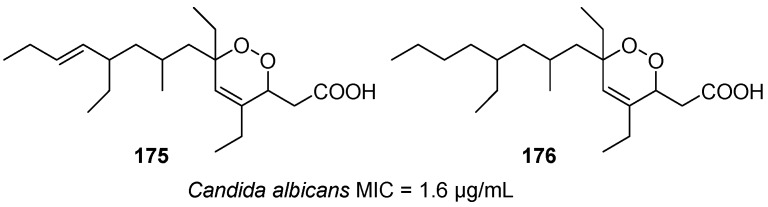
Antifungal natural 1,2-dioxenes **175**, **176**.

In 1990, Gunasekera with colleagues isolated 1,2-dioxenes **175** and **176** from sea sponge *Plakortis angulospiculatus* (Scheme 27) [120]. It was shown that these natural peroxides exhibit antifungal activity against *Candida albicans* (MIC = 1.6 µg/mL).

**Scheme 28:**
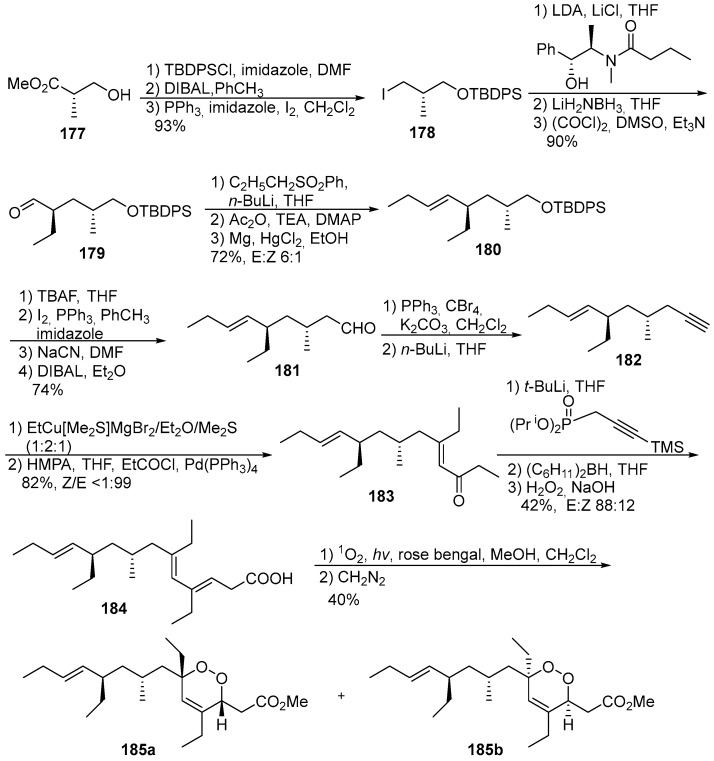
Synthesis of stereoisomeric 1,2-dioxenes **185**.

The total synthesis of stereoisomeric 1,2-dioxenes **185** was performed in 18 steps with a total yield of 2.8% (Scheme 28) [121]. The treatment of hydroxy ester **177** with *tert*-butyldiphenylsilyl chloride followed by DIBAL reduction and replacement of hydroxyl by iodine provided iodide **178**, which was converted into aldehyde **179** by subsequent asymmetric alkylation, reduction of prepared amide into alcohol and Swern oxidation. Alkene **180** was synthesized from sulfone derivative of aldehyde **179** to obtain mainly *trans*-isomer. Deprotection of **180**, nucleophilic substitution of hydroxyl by iodine, subsequent substitution of iodine by cyano-group and reduction of cyano-group led to aldehyde **181** which was then transformed into alkyne **182** by PPh_3_/CBr_4_ treatment and HBr elimination. The synthesis of enone **183** was performed by addition of ethylcuprate followed by propionyl chloride to alkyne **182**. The diene **184** was prepared predominantly in *trans*-conformation by condensation of enone **183** with lithium salt of propargyl phosphonate with subsequent hydroboration. Ene reaction of diene **184** with singlet oxygen and methylation by diazomethane resulted in 1,2-dioxenes **185а** and **185b**.

**Scheme 29:**
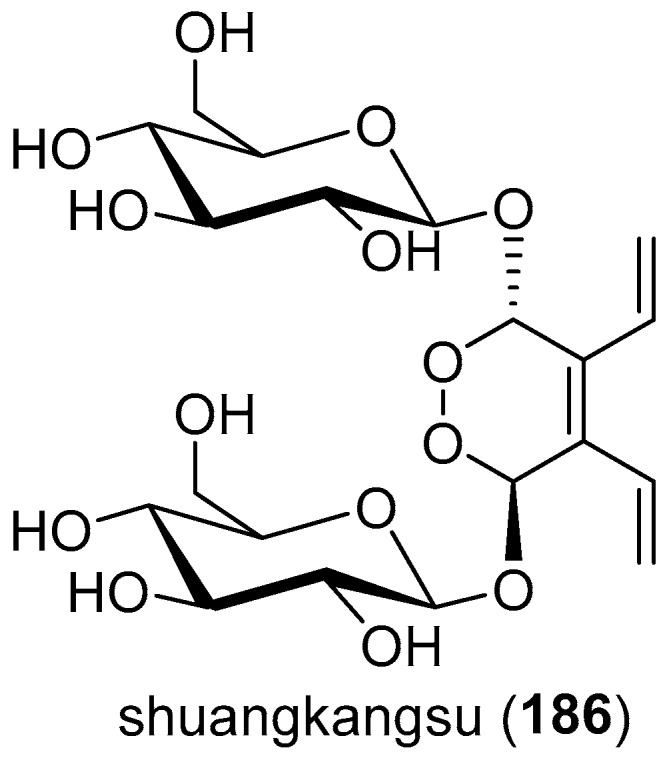
Peroxide shuangkangsu (**186**) isolated from *Lonicera japonica*.

The cyclic peroxide shuangkangsu (**186**) isolated from the buds of *Lonicera japonica* showed high antiviral activity against respiratory syncytial virus on the cell lines and influenza virus in the chicken embryos (Scheme 29) [122].

**Scheme 30:**
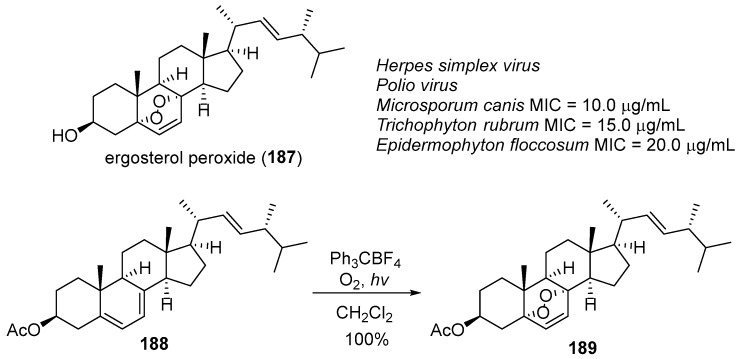
Ergosterol peroxide (**187**), exhibited antiviral and antifungal activities and synthesis of its derivatives.

Ergosterol peroxide (**187**) isolated from different natural sources including the fungi *Pycnoporus cinnabarinus* demonstrated moderate antiviral activity against *Herpes simplex* и *Polio* virus [123], and also antifungal activity against pathogenic fungi *Microsporum canis*, *Trichophyton rubrum* and *Epidermophyton floccosum* (Scheme 30) [124]. Ergosterol peroxide acetate **189** was synthesized with quantitative yield by photo-oxidation of ergosterol acetate **188** in the presence of trityl tetrafluoroborate (Scheme 30) [125].

**Scheme 31:**
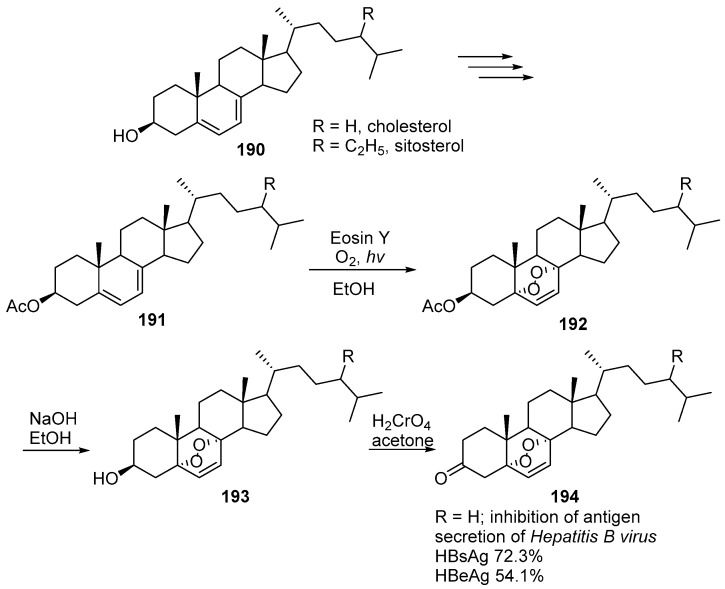
Synthesis of **194**, antiviral analogs of ergosterol peroxide.

Analogs of ergosterol peroxide without multiple bonds in the side chain **194** were obtained by eosin Y catalyzed photooxidation of steroids **191** followed by hydrolysis and oxidation. Peroxides **194** showed significant ability to inhibit the grown of hepatitis B virus (Scheme 31) [126].

**Scheme 32:**
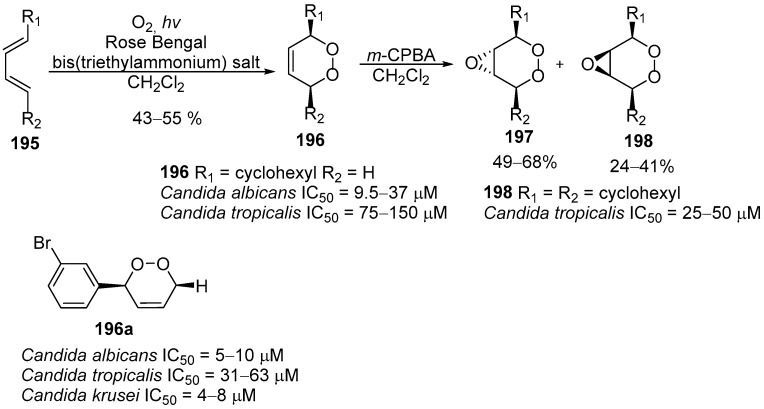
Synthesis of 1,2-dioxenes **196** and their further modification.

A number of 1,2-dioxenes **196** with simpler structure were synthesized from 1,3-butadienes **195**. Epoxidation of **196** resulted in 1,2-dioxanes **197** and **198**. These peroxides exhibit moderate antifungal activity against *Candida* family (Scheme 32) [127]. Later, a wide range of derivatives **196**, **197** and **198** that inhibits *Candida albicans* was synthesized [128]. 1,2-Dioxene **196а** showed high antifungal activity against *C. tropicalis* and *C. krusei* [129].

## 6. 1,2,4-Trioxanes

Among the class of 1,2,4-trioxane, various aspects of the biological activity of artemisinin and its derivatives are studied most extensively. Synthetic strategies for peroxide ring construction in artemisinin were discussed in detail [130]. Compared with other methods the synthesis of artemisinin (**1**) based on dihydroartemisinic acid seems most preferable [131], as it can satisfy the demand for cheaper production of sufficient quantities of artemisinin. The key stages of the transformation of dihydroartemisinic acid into artemisinin (**1**) are described in the fundamental studies of Richard K. Haynes [132].

**Scheme 33:**
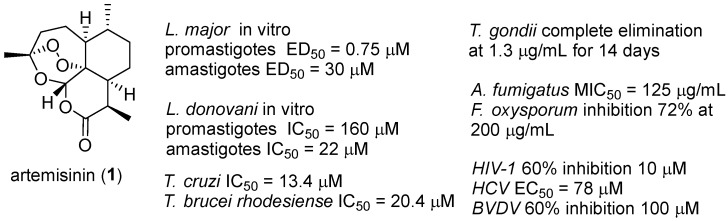
Artemisinin and various types of its bioactivity.

In addition to antimalarial and cytotoxic activity, artemisinin (**1**) has activity against trypanosomatides *Leishmania major* [133], *Leishmania donovani* [134], *Trypanosoma brucei rhodesiense* and *Trypanosoma cruzi* [135], as well as parasite *Toxoplasma gondii* (Scheme 33) [136]. Artemisinin showed a synergistic or additive effect in combination with itraconazole against fungi *Aspergillus fumigatus* [137], as well as moderate activity against *Fusarium oxysporum* [138]. Antiviral activity of artemisinin (**1**) was reported in few studies; inhibition of the human immunodeficiency virus (HIV-1) at 60% in peripheral blood mononuclear cells [139], the hepatitis C virus (HCV) in human liver cells [140], the bovine viral diarrhea virus (BVDV) [141], as well as hepatitis B virus [142] was shown.

**Scheme 34:**
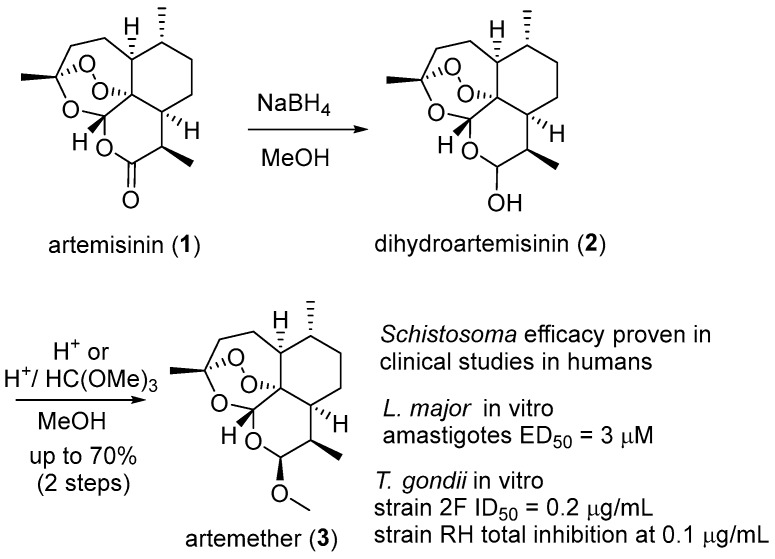
Synthesis of artemether (**3**) and its bioactivity.

Artemisinin derivative, artemether (**3**), is actively used to treat schistosomiasis, a parasitic disease caused by flat worms of the genus *Schistosomiasis* [46,47]. A double-blind field trial in the Poyang Lake region (southern China) confirmed that artemether (**3**) significantly reduces the frequency and intensity of *S. japonicum* infection and does not cause side effects [143]. Despite the proven pathogenic effects on the reproductive system of *Fasciola hepatica* [144,145], artemether (**3**) had practically no effect in the treatment of fascioliasis in humans [146]. Artemether activity against *Leishmania major* [133], and *Toxoplasma gondii* [136,147] was detected; it was shown also that artemether is effective in the treatment of experimental rheumatoid arthritis [148,149]. Artemether (**3**) is obtained by reduction of artemisinin (**1**) to dihydroartemisinin (**2**) followed by methylation (Scheme 34) [150,151]. This synthesis can be performed in flow reactor [152,153].

**Scheme 35:**
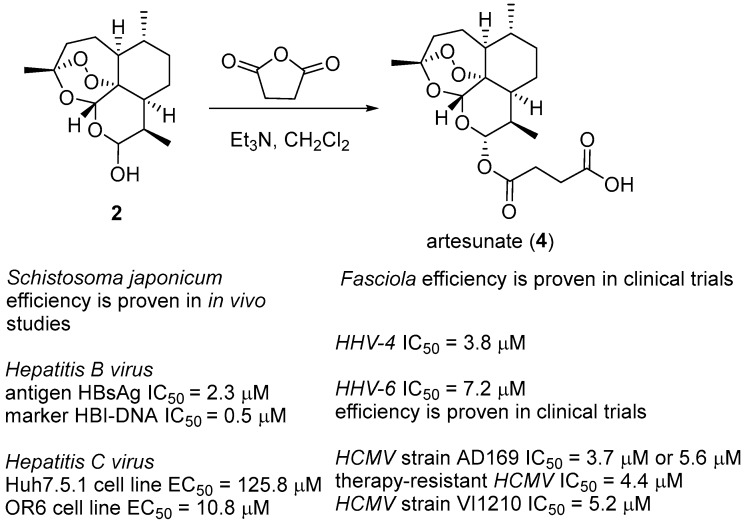
Synthesis of artesunate (**4**) and its bioactivity.

Artesunate (**4**), artemisinin derivative containing free carboxylic group showed high efficiency against *S. japonicum* [154,155], and also in the therapy of fascioliasis caused by *Fasciola hepatica* or *Fasciola gigantica* (Scheme 35) [156]. It was determined that artesunate (**4**) causes changes in the reproductive system of *Fasciola hepatica* [144]. Antiviral activity of artesunate is displayed against the hepatitis В virus [157] and the hepatitis C virus [158], the herpes virus (HHV) type 4 and type 6 [159,160], human cytomegalovirus (HCMV) [161,162,163], including therapy-resistant mutants of human cytomegalovirus [164]. Artesunate (**4**) is prepared by reaction of dihydroartemisinin (**2**) with succinic anhydride in basic conditions (Scheme 35) [152].

**Scheme 36:**
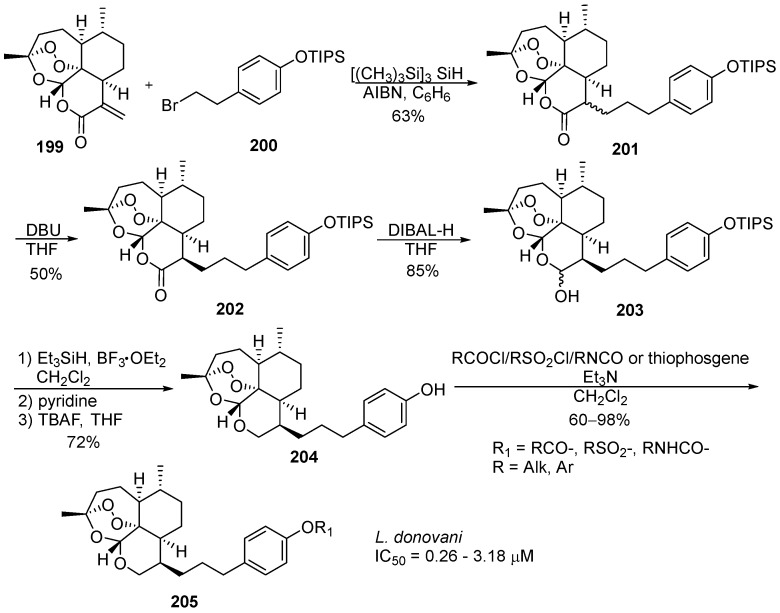
Antileishmaniasis derivatives of artemisinin **205**.

The library of 10-deoxo-derivativies of artemisinin **205** showed high activity against parasites *Leishmania donovani* (Scheme 36). Synthesis of **205** was performed from artemisitene **199** by radical addition of compound **200** followed by reduction of carbonyl group in **202**. Dehydration and deprotection resulted in phenol **204**, which formed a series of compounds **205** by reaction with derivatives of carboxylic and sulfonic acids [165].

Attempts to synthesize the antitoxoplasma derivatives of artemisinin have been made [147,166], however their in vitro activity did not exceed the activity of artemether (**3**).

**Scheme 37:**
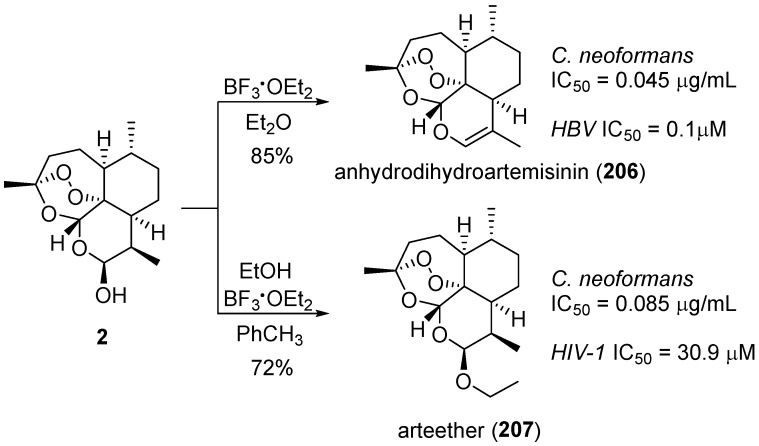
Antifungal and antiviral artemisinin derivatives— anhydrodihydroartemisinin (**206**) and arteether (**207**).

Among artemisinin derivatives tested for fungicidal activity, anhydrodihydroartemisinin (**206**) and arteether (**207**) were most active against *Cryptoccocus neoformans* [167], their activity surpassed the one of amphotericin B. Both derivatives were obtained [168,169] in one step from dihydroartemisinin (**2**) (Scheme 37). Later it was found that arteether (**207**) [170] exhibits moderate antiviral activity against human immunodeficiency virus and anhydrodihydroartemisinin (**206**) [171] is high active against hepatitis B virus.

**Scheme 38:**
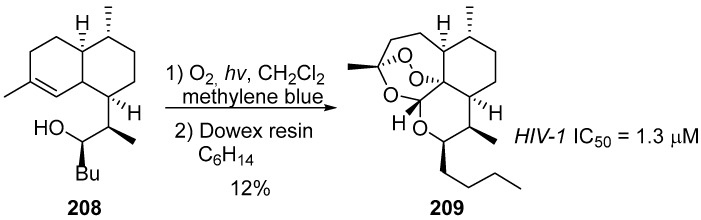
Antiviral artemisinin derivative **209**.

High antiviral activity against human immunodeficiency virus [170] was demonstrated by butyl-derivative of artemisinin **209**, prepared via photo-oxidation of alcohol **208** with 12% yield (Scheme 38) [172].

**Scheme 39:**
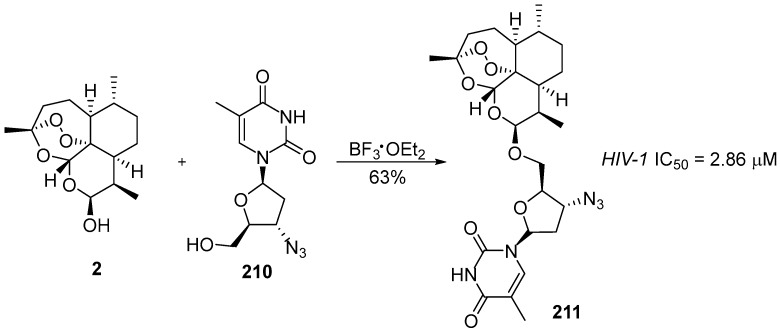
Synthesis of hybrid **211** based on dihydroartemisinin **2** and azidothymidine **210**.

Combination of dihydroartemisinin (**2**) with antiviral drug azidothymidine (**210**) resulted in compound **211** exhibited both antimalarial as antiviral activity (Scheme 39) [173].

**Scheme 40:**
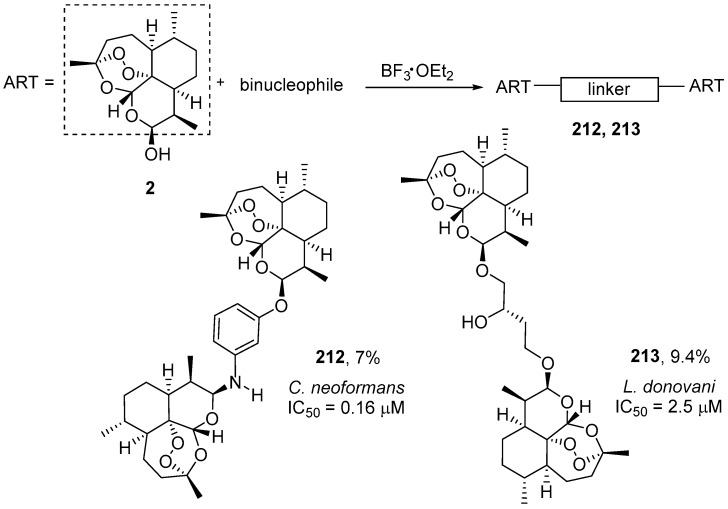
Synthesis of artemisinin dimers **212** and **213** with antifungal and anti-parasitic activity.

A new trend in the medical chemistry of artemisinin derivatives is the synthesis of dimers and trimers. Among a series of artemisinin dimers obtained by condensation of dihydroartemisinin (**2**) with binucleophile, compounds **212** and **213** showed the highest activity against fungi *Cryptococcus neoformans* and parasites *Leishmania donovani,* respectively (Scheme 40) [174].

Dimers and trimers of artemisinin showed antiviral activity were summarized in Table 1.

**Scheme 41:**
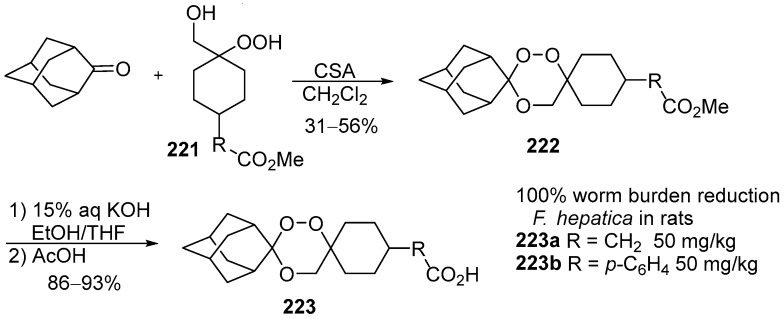
Anthelminthic activity of synthetic 1,2,4-trioxanes **223**.

Synthetic 1,2,4-trioxanes **223** obtained by condensation of hydroxy-hydroperoxide **221** with adamantane followed by hydrolysis of ester **222** showed high in vivo activity against helminths *Fasciola hepatica* in rats (Scheme 41) [188].

## 7. 1,2,4,5-Tetraoxanes

**Scheme 42:**
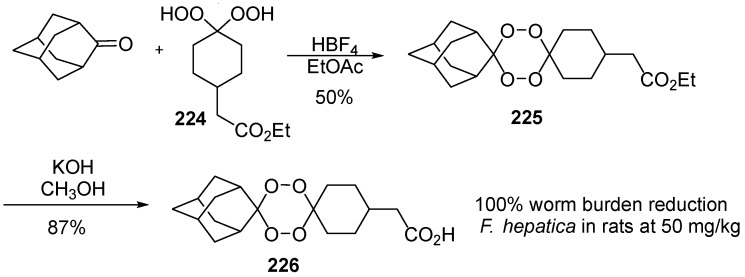
Synthesis of 1,2,4,5-tetraoxane **226** with high anthelmintic activity.

Synthetic 1,2,4,5-tetraoxane **226** [189], prepared by condensation of *bis*-hydroperoxide **224** with adamantanone followed by hydrolysis ester **225**, showed high in vivo activity against helminths *Fasciola hepatica* in rats (Scheme 42) [188].

**Scheme 43:**
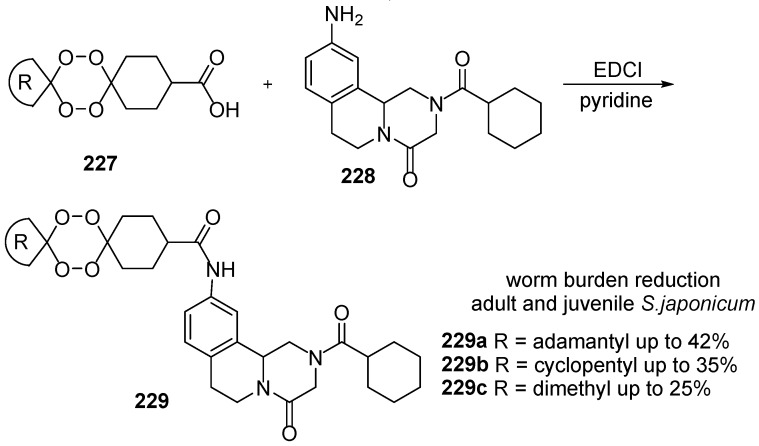
Synthesis of hybrids **229** as anti-schistosomal agents.

Based on related 1,2,4,5-tetraoxanes **227**, hybrids **229** with a fragment of an anthelmintic drug praziquantel **228** were obtained (Scheme 43) [190]. Synthesized hybrids **229** exhibit high activity against *Schistosoma japonicum* and *Schistosoma mansoni* [191].

**Scheme 44:**
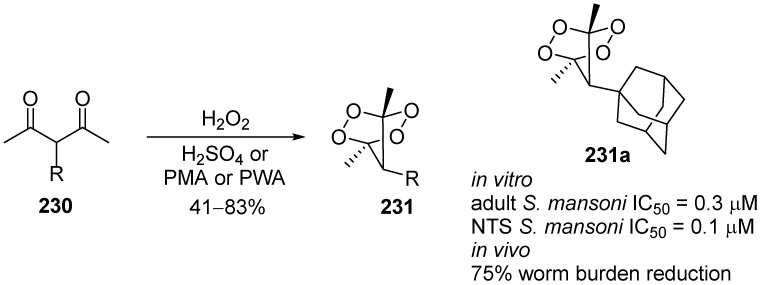
Synthesis of bridged 1,2,4,5-tetraoxanes **231**.

Bridged 1,2,4,5-tetraoxanes **231** demonstrated high in vitro and in vivo activity against trematodes *Schistosoma mansoni* [74,192]. The best result was shown for adamantane-substituted tetraoxane **231a** which caused 75% worm burden reductions in *S. mansoni* harbored in mice following the administration of peroxide at single oral dose of 400 mg/kg. Peroxides **231** were synthesized from β-diketones **230** and H_2_O_2_ with good yields by the action of various acids: sulfuric [193], phosphomolybdic and phosphotungstic acids [194] (Scheme 44).

## 8. Acyclic Peroxides

Many acyclic peroxides are applied as oxidants in organic synthesis [195,196]. Benzoyl peroxide (**234**) is actively used in the food industry as a flour bleach [197,198,199], and in pharmaceuticals. The first mention about the medical using of benzoyl peroxide dates back to 1929, where Lyon and Reynolds reported effective treatment of burns, wounds and varicose veins by benzoyl peroxide [200]. Subsequently it was found that it has antibacterial [201,202], anti-inflammatory [203], cheratolic [204], and wound-healing [205,206] effects. Presently, benzoyl peroxide is widely used agent for acne treatment because of its efficacy and good tolerability in patients [207,208]. Benzoyl peroxide is a good alternative to monotherapy with antibiotics for the treatment of *Acne vulgaris* caused antibiotic-resistant strains, for example *Propionibacterium acnes* [209,210].

**Scheme 45:**
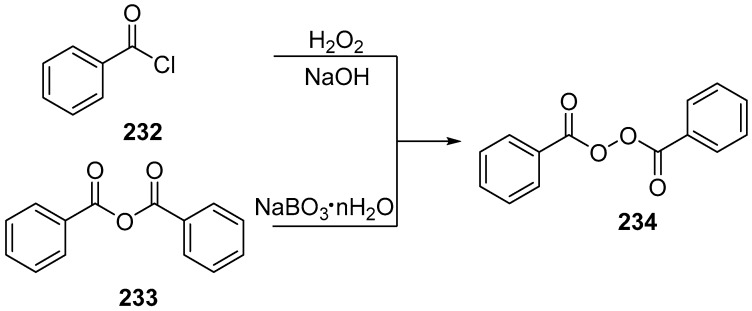
Benzoyl peroxide (**234**) production.

Benzoyl peroxide is commercially produced by the reaction of benzoyl chloride (**232**), sodium hydroxide and hydrogen peroxide (Scheme 45, top) [211,212]. Benzoyl peroxide can also be prepared from benzoic anhydride (**233**) by the action of an alkali metal perborate in an aqueous solution (Scheme 45, bottom) [213].

**Scheme 46:**
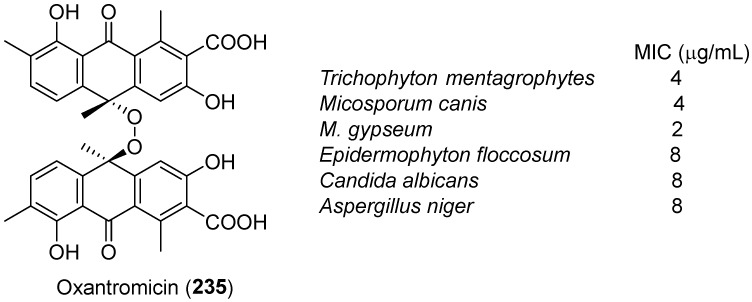
Antifungal natural acyclic peroxide, oxanthromicin (**235**).

Oxanthromicin (**235**) isolated from bacteria *Actinomadura* sp. [214] showed high antifungal activity against *Trichophyton mentagrophytes*, *Micosporum canis*, *M. gypseum*, *Epidermophyton floccosum*, *Candida albicans*, and *Aspergillus niger* (Scheme 46) [215].

**Scheme 47:**
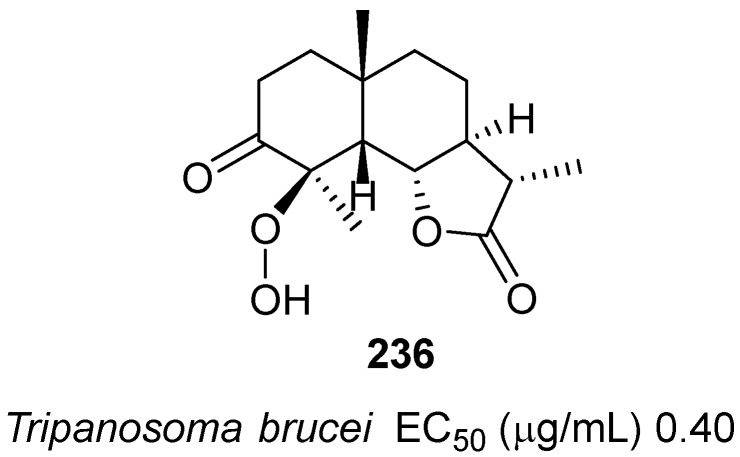
Anti-trypanosomal hydroperoxide **236**.

Hydroperoxide **236** obtained by biotransformation of *Artemisia cina* metabolites exhibits high in vitro activity against protozoa *Trypanosoma brucei* (Scheme 47) [216].

**Scheme 48:**
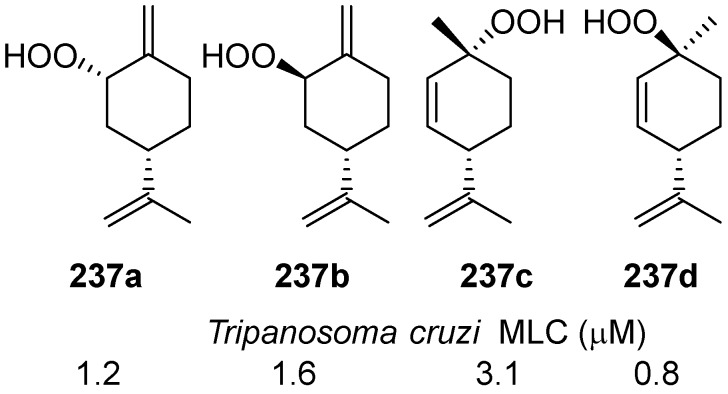
Hydroperoxides **237a**–**d** isolated from *Chenopodium ambrosioides*.

Four monoterpene hydroperoxides **237a**–**d** were isolated from aerial parts of *Chenopodium ambrosioides*. Minimum lethal concentration (MLC) in vitro of its peroxides against *Trypanosoma cruzi* was 1.2, 1.6, 3.1, and 0.8 µM, respectively (Scheme 48) [107].

**Scheme 49:**
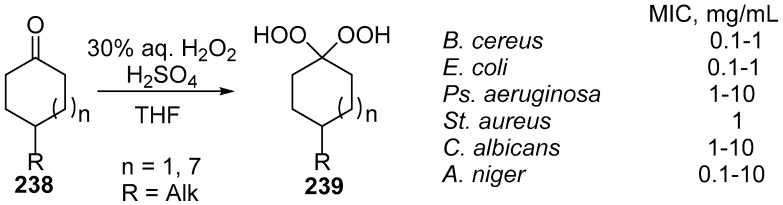
Antimicrobial and antifungal geminal *bis*-hydroperoxides **239**.

A geminal *bis*-hydroperoxides **239** synthesized from cyclic ketones **238** show pronounced in vitro antimicrobial and antifungal activity against *B. cereus*, *E. coli*, *P. aeruginosa*, *S. aureus*, *C. albicans*, and *A. niger* comparable with the effect of some antiseptics and, to a lesser extent, antibiotics (Scheme 49) [217].

## 9. Conclusions

The biological activity of organic peroxides is usually associated with the antimalarial properties of artemisinin and its derivatives. However, the analysis of published data indicates that organic peroxides exhibit a variety of biological activity—anthelmintic, antifungal, antiviral, etc.—which is still being given insufficient attention.

Efforts of synthetic chemists are currently directed at the development of methods for the synthesis of biologically active natural peroxides, the modification of natural peroxides and the search of synthetic peroxides, which are not inferior to their natural and semisynthetic analogs, but are substantially cheaper. It seems that progress in the synthesis of biologically active peroxides will be mainly related to the last two directions.

In view of very dynamic development of these areas of medical chemistry, in the near future, one should expect a breakthrough in the synthesis of biologically active peroxides and in understanding of its action with respect to a wide range of bio-targets.

## Figures and Tables

**Table 1:** Dimers and trimers of artemisinin showed antiviral activity.

No.	Dimer/Trimer Structure	Synthesis	Bioactivity
1	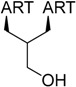 **214**	from artemisinin (**1**) 3 steps, 67% [175]	*HCMV* EC_50_ = 0.15 µM [176,177]
2	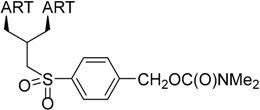 **215**	from **214** 3 steps, 42% [178]	*HCMV* EC_50_ = 0.06 µM [176]
3	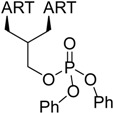 **216**	from **214** 1 step, 96% [179]	*HCMV* EC_50_ = 0.04 µM [180,181,182]
4	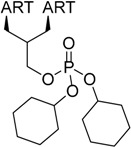 **217**	from **214** 1 step, 25% [183]	*HCMV* EC_50_ = 44 nM [183]
5	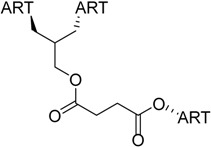 **218**	from *threo*-**214** and artesunate (**4**), 1 step, quantitative yield [184]	*HCMV* EC_50_ = 0.04 µM [184,185]
6	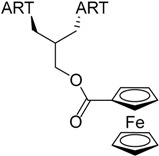 **219**	from *threo*-**214** 1 step, 67% [186]	*HCMV* EC_50_ = 0.11 µM [186]
7	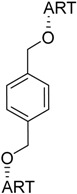 **220**	not reported	*HCV* EC_50_ = 3.2 µM [187]
